# Ozonized biochar filtrate effects on the growth of *Pseudomonas putida* and cyanobacteria *Synechococcus elongatus* PCC 7942

**DOI:** 10.1186/s40643-021-00491-2

**Published:** 2022-01-06

**Authors:** Oumar Sacko, Nancy L. Engle, Timothy J. Tschaplinski, Sandeep Kumar, James Weifu Lee

**Affiliations:** 1 Department of Chemistry and Biochemistry, Old Dominion University, Norfolk, VA 23529 USA; 2 Oak Ridge National Laboratory, PO Box 2008, Oak Ridge, TN 37831 USA; 3 Department of Civil and Environmental Engineering, Old Dominion University, Norfolk, VA 23529 USA

**Keywords:** *Pseudomonas putida*, Ozonized biochar filtrate, Dissolved organic carbon, Bioassay, Biological effects of ozonized biochar substances

## Abstract

**Background:**

Biochar ozonization was previously shown to dramatically increase its cation exchange capacity, thus improving its nutrient retention capacity. The potential soil application of ozonized biochar warrants the need for a toxicity study that investigates its effects on microorganisms.

**Results:**

In the study presented here, we found that the filtrates collected from ozonized pine 400 biochar and ozonized rogue biochar did not have any inhibitory effects on the soil environmental bacteria *Pseudomonas putida,* even at high dissolved organic carbon (DOC) concentrations of 300 ppm. However, the growth of *Synechococcus elongatus* PCC 7942 was inhibited by the ozonized biochar filtrates at DOC concentrations greater than 75 ppm. Further tests showed the presence of some potential inhibitory compounds (terephthalic acid and *p*-toluic acid) in the filtrate of non-ozonized pine 400 biochar; these compounds were greatly reduced upon wet-ozonization of the biochar material. Nutrient detection tests also showed that dry-ozonization of rogue biochar enhanced the availability of nitrate and phosphate in its filtrate, a property that may be desirable for soil application.

**Conclusion:**

Ozonized biochar substances can support soil environmental bacterium *Pseudomonas putida* growth, since ozonization detoxifies the potential inhibitory aromatic molecules.

**Graphical Abstract:**

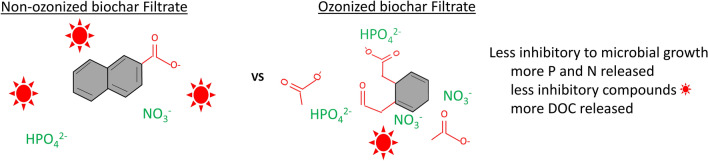

**Supplementary Information:**

The online version contains supplementary material available at 10.1186/s40643-021-00491-2.

## Introduction

The pyrolysis of biomass to biochar may produce some chemical compounds such as polyaromatic hydrocarbons (PAH’s), furans, and dioxins that may be toxic to microorganisms (Lyu et al. 2016). The use of biochar was proposed for the remediation of soil and waste waters (Li et al. 2017; Wang et al. 2015). Before being applied to soils, the toxicity effects of biochar on microorganisms needs to be investigated. Several factors including the feedstock used for pyrolysis, the temperature of pyrolysis, and the age of biochar may affect its toxicity (Lehmann and Joseph 2009; Hale et al. 2012). Smith et al. (2016) showed that the water soluble organic compounds from pinewood biochars made at high temperatures (above 400 °C) exhibit less toxicity on cyanobacteria compared to pinewood biochars made at lower temperatures (below 400 °C).

The effect of ozonization was shown to create some oxygen functional groups on the biochar surface, which resulted in an increase in cation exchange capacity (CEC) (Sacko et al. 2020; Huff et al. 2018), a key property for fertilizer retention in soil; we recently showed the biochar CEC increased by up to almost 10 times upon ozonization under dry conditions (Kharel et al. 2019). This type of ozonized biochar materials could potentially have significant applications for agroecosystem sustainability such as to help unlock phosphorus from certain insoluble phosphate materials and improve soil properties. However, to consider the large-scale applications of ozonized biochar with soils, it is important to study the potential impacts of ozonized biochar substances on soil microorganisms. Among the various soil microorganisms, we are interested in *P. putida*, a common bacterium in the plant rhizosphere. The presence of *P. putida* in soil was shown to promote plant growth (Silby et al. 2011; Mercado-Blanco and Bakker 2007). Recently, there has been particular interest in the inoculation of biochar with bacteria prior to its introduction to soils and plant roots (Tu et al. 2020). Some bacteria-inoculated biochars were shown to improve the soil microbial community and plant growth (Wei et al. 2020; Głodowska et al. 2017; Egamberdieva et al. 2018). Our ozonized biochar may be a potential candidate for the development of bacteria-inoculated biochars; given the increased oxygen functional groups on its surface (Sacko et al. 2020; Kharel et al. 2019), we would expect it to immobilize nutrients that can be used by soil bacteria. In addition, our ozonized biochar was shown to unlock phosphate from insoluble hydroxyapatite material (Sacko et al. 2020), which would enable plant roots to have easier access to usable phosphate. However, before considering the application of ozonized biochar as a bacterial inoculant, it is particularly important to test whether the latter would be toxic or not to microbial growth. To the best of our knowledge, there have been no studies on the effects of ozone-treated biochar on microorganisms. In this study, the effects of ozonized biochar water extractable dissolved organic carbon were tested on the growth of *Pseudomonas putida,* which is a soil environmental bacterium*.*

Freshwater on the land surface is an essential part of the water cycle. Allochthonous organic matter can gain access to the watershed via soil leaching. If ozonized biochar were to be applied to soil systems, the soil leaching would bring some ozonized biochar molecular fragments, such as the dissolved organic carbon materials, into the freshwater. Therefore, it is also important to know what effects the ozonized biochar filtrate would have on microorganisms of a freshwater ecosystem. In addition, contamination of freshwater by toxic metals poses serious concerns (Zhong et al. 2018). Therefore, for the application of ozonized biochar as a remediation of toxic metals in freshwater to be considered, it is important to determine its effects on freshwater microorganisms. Cyanobacteria are commonly found in freshwater. In this study, we also investigated the effects of ozonized biochar water soluble organic materials (filtrate) on *Synechococcus elongatus* PCC 7942, a freshwater cyanobacteria. Finally, we tested for the potential differences between the filtrates of ozonized biochars and non-ozonized biochar.

## Materials and methods

### Biochar materials, ozonization treatments, and dissolved organic carbon measurements

The pine biochars used for this study were produced in a similar fashion as previously reported (Sacko et al. 2020) with some pre-pyrolysis changes. The pre-pyrolysis preparation here involved the removal of the bark, the chopping of the pinewood into smaller pieces (5 mm diameter) and the washing of 50 g of the pine biomass with 300 mL of milli-Q water to reduce ash content (Cen et al. 2016; Deng et al. 2013). The washed pinewood was then dried prior to its pyrolysis. The pinewood was pyrolyzed under atmospheric pressure to reach the highest treatment temperatures of 300 °C, 400 °C, or 500 °C. The resulting biochars obtained were named pine 300 or P300 (40.98 ± 1.44% yield), pine 400 or P400 (28.36 ± 0.28% yield), and pine 500 or P500 (25.10 ± 1.30% yield). In addition, rogue biochar (RBC) from Oregon Biochar Solutions was also used. This biochar was made from a mixture of softwood tree materials, such as pine and Douglas fir through pyrolysis at 700 °C; more characteristics about the Rogue biochar can be found in a previous report (Kharel et al. 2019). All biochars were ground and sieved through a 106-µm screen and stored in an oven at 105 °C.

Each biochar was ozone treated under wet or dry conditions as described in our previous work (Sacko et al. 2020) with a slight modification. For the wet ozone treatment, 1.5 g of oven-dried biochar was mixed with 10 mL of milli-Q water. The mixture was ozonized for 90 min in a tubular reactor using an oxygen gas stream containing ozone at a gas flow rate of 3 L/min from a Welsbach T-series ozone generator. Pure oxygen at 8 psi was fed into the ozone generator that used a voltage of 115 V for its corona discharge to convert a significant amount of oxygen molecules to ozone molecules. After the ozone treatment, the 10 mL filtrate was collected using a Büchner vacuum filtration system with Fisherbrand P8 filter paper (catalog number 09-795B). The biochar was subsequently washed with 25 mL and 300 mL of milli-Q water and the resulting filtrates were collected for each wash. The washed biochar was then dried in the oven at 105 °C. The dry-ozone treatment was conducted in a similar manner, but here, no water was added to the biochar prior to the ozone treatment; after 90 min of ozone treatment, the biochar was subsequently washed with 10 mL, 25 mL, and 300 mL of milli-Q water. The filtrate was collected for each wash. The washed biochar was dried in the oven at 105 °C. The non-ozonized biochar control was prepared in a similar manner, but without the ozonization; briefly 1.5 g of biochar was washed with 10 mL, 25 mL, and 300 mL of milli-Q water. The filtrates were collected, and the washed biochar was stored in the oven at 105 °C.

The filtrates that were collected contained water extractable organic carbon materials. In an effort to quantify the dissolved organic carbon (DOC) in these filtrates, they were further filtered through a 0.2-µm pore-size filter. The concentration of the dissolved organic carbon in the filtrate and the pH of the biochar slurry were measured in a similar manner, as described in our previous work (Sacko et al. 2020). Note that a 0.2-µm filtration also ensures removal of potential bacterial contaminants, prior to the use of the filtrate for the toxicity assay.

### Toxicity assay of biochar filtrate on *Pseudomonas putida* and *Synechococcus elongatus* PCC 7942

Wild-type cyanobacteria *Synechococcus elongatus* PCC 7942 were taken from log-phase growth and inoculated into fresh BG-11 liquid medium buffered at pH 8.0 with Tris Ethylenediaminetetraacetic acid (TES). Similarly, the wild-type *Pseudomonas putida* KT2440 cells were inoculated into fresh Luria Broth (LB) medium buffered with Tris/Tris HCl at pH 7.0. These two cultures served as the stock, and they were each shaken prior to inoculation of the wells. The bioassay setup was performed with a similar concept as done by Smith et al. (2016) with some modifications. The assay was conducted with Corning Costar 24-well plates. The total volume of liquid loaded in each well was 2500 µL. The biochar filtrate was loaded into each well to achieve a DOC concentration ranging from 0 ppm (no DOC control) to 300 ppm; the biochar filtrate (0–1000 µL) was mixed with milli-Q water (0–1000 µL) so that the combined volume did not exceed 1000 µL. To bring the total volume up to 2500 µL, 1500 µL of the *S. elongatus* PCC 7942 or *P. putida* in BG-11 or LB medium, respectively, were added to each well. The cells were pipetted from the stock liquid cultures; the stock cultures were hand shaken vigorously prior to each transfer to the wells to ensure that the original cell concentration was similar across the wells. If the desired DOC concentration was not able to be obtained due to the low DOC concentration, the well was left empty. The blank wells consisted of just 1500 µL of BG-11 or LB medium without the cells. The setup of the plates is shown in Table 1. The plates inoculated with *P. putida* were incubated at 37 °C and the plates inoculated with *S. elongatus* PCC 7942 were incubated at room temperature under actinic light intensity 15–20 µmol/m^2^/s.

**Table 1:** Setup of the bioassay multi-well plates

	1	2	3	4	5	6
A	1500 µL cells + 300 ppm (non-ozonized)	1500 µL cells + 150 ppm (non-ozonized)	1500 µL cells + 75 ppm (non-ozonized)	1500 µL cells + 25 ppm (non-ozonized)	1500 µL cells + 10 ppm (non-ozonized)	1500 µL cells + 2 ppm (non-ozonized)
B	1500 µL cells + 300 ppm (dry-ozonized)	1500 µL cells + 150 ppm (dry-ozonized)	1500 µL cells + 75 ppm (dry-ozonized)	1500 µL cells + 25 ppm (dry-ozonized)	1500 µL cells + 10 ppm (dry-ozonized)	1500 µL cells + 2 ppm (dry-ozonized)
C	1500 µL cells + 300 ppm (wet-ozonized)	1500 µL cells + 150 ppm (wet-ozonized)	1500 µL cells + 75 ppm (wet-ozonized)	1500 µL cells + 25 ppm (wet-ozonized)	1500 µL cells + 10 ppm (wet-ozonized)	1500 µL cells + 2 ppm (wet-ozonized)
D			Blank No cells 1500 µL LB/BG11 + 1000 µL milli-Q	Blank No cells 1500 µL LB/BG11 + 1000 µL milli-Q	0 ppm DOC 1000 µL milli-Q + 1500 µL cells	0 ppm DOC 1000 µL milli-Q + 1500 µL cells

The first three rows show the DOC concentrations (ppm) of the biochar filtrates from low to high concentration (right to left). The filtrates from the non-ozonized biochar, dry-ozonized biochar, and wet-ozonized are represented in row A, B, and C, respectively. The wells labeled “0 ppm DOC” are the controls with no DOC, and the wells labeled “Blank” contain the growth medium without cells

The assay was performed using the filtrates from RBC and P400 biochars. The growth of the cells was monitored by measuring the optical density (OD730) using a BioTek Synergy HT multimode microplate reader at absorbance measuring light wavelength of 730 nm on day 0, day 0.5, day 1, day 2, day 3, day 4, and day 5 for the *P. putida* assays. Note that this optical density is a measurement of cell culture population density by the light scattering effect of the microbial cells. 730 nm was preferred to 600 nm as longer wavelengths have less interference issues (Hecht et al. 2016). For that purpose, in this project, OD730 was used to reduce absorbance signals from the biochar filtrates. For the PCC 7942, the optical density was measured at 730 nm every 2 days from day 0 to day 16. Before each measurement, if evaporation occurred, milli-Q water was added to replenish wells to the initial liquid level. Cells from wells were mixed by pipetting prior to each measurement. Photographs of the multi-well plates were also taken prior to each measurement. Each plate assay was done in duplicate (*n* = 2).

As a background control, OD730 measured on day 0 for each well was used as the blank for that well for the entire time of the growth assay. For that matter, the OD730 at day 0 was subtracted from the OD730 values of the same plate at days 2, 4, 6, 8, 10, 12, 14, and 16. The purpose of that was to just monitor the OD730 due to the growth of cells and not the scattering that may be due to biochar DOC particles or other interferences.

Another background control was performed to see if biochar filtrate varied over time; an assay was also performed using just the filtrates at the different DOC concentrations without bacterial cells (Additional file 1: Fig. S1). The purpose for that is because the biochar filtrates also display a wide range of absorbance that may change over time. These plates were observed over the period of the growth assay, and their OD730 were also recorded to see if any change occurred in filtrates when bacterial cells were absent. Each control plate assay was done in duplicate (*n* = 2). The OD730 recorded for these controls did not vary over the time of the study.

### Determination of the anions released by biochar using ion chromatography

To determine the effect of biochar ozonization on nutrient content, the concentrations of several anions (nitrate, phosphate, sulfate, and chloride) were measured from the filtrate of ozonized biochars and the filtrate of the non-ozonized biochars. A Dionex 5000 Ion chromatography instrument packed with AG23/AS23 column of 0.4 × 250 mm was used for anion separation. 4.5 mM Na_2_CO_3_ and 0.8 mM NaHCO_3_ solutions were used as mobile phase eluents. The samples were loaded on a Dionex AS 40 autosampler. The signals were detected with a conductivity detector. The data collection time of the ion chromatography was set to 30 min. A Thermo Scientific Dionex 7 Anion standard solution (Thermo Fisher 057,590) was used to quantify the anions. For this analysis, the filtrate was collected from the 2nd wash (25 mL wash) of the 1.5 g biochar. Each sample type was measured in triplicate (*n* = 3). A standard acetic acid ACS grade was also used as a reference.

### Identification of potential bacterial inhibitory compounds by mass spectrometry

The presence of potential inhibitory compounds was tested in the filtrate of the ozonized biochars and the non-ozonized biochar. Aliquots of filtrate were dried under an inert stream of nitrogen to give approximately 15 mg of DOC content. The dried filtrates were then dissolved in 500 µl silylation grade acetonitrile followed by trimethylsilyl derivatization with 500 ul *N*-Methyl-*N*-trimethylsilyltrifluoroacetamide plus 1% 2,2,2-Trifluoro-*N*-methyl-*N*-(trimethylsilyl)-acetamide, Chlorotrimethylsilane (MSTFA + 1% TMCS) and heated at 70 °C for 1 h. After 2 days, 1 µL was injected to an Agilent 7890A-5975C inert XL gas chromatography–mass spectrometry (GC–MS) system using previously described conditions (Tschaplinski et al. 2012). Inhibitory compounds were identified using a Wiley Registry 10^th^ Edition/NIST 2014 Mass Spectral library.

## Results and discussion

### Dissolved organic carbon in the filtrates of ozonized biochars

**Fig. 1:**
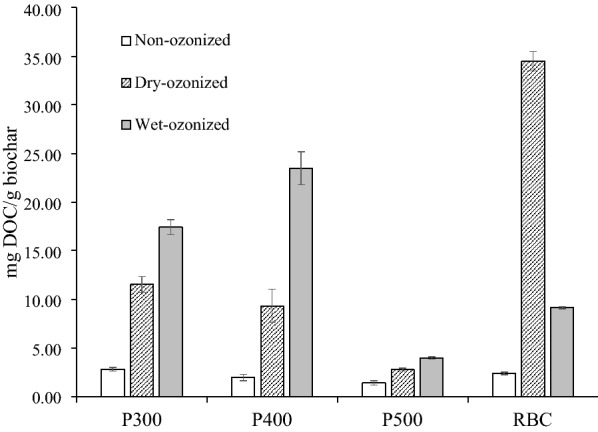
Total dissolved organic carbon extracted from the pine 300 (P300), pine 400 (P400), pine 500 (P500), and rogue biochar (RBC) before and after wet/dry ozonization. The DOC amounts reported are in mg DOC/g biochar. The error bars denote the error of the average of 3 measurements (*n* = 3)

The dissolved organic carbon (DOC) concentration of the biochar filtrates was measured before and after ozonization. The pristine pine biochars (P300, P400, P500) showed less DOC in their filtrate as the pyrolysis temperature increased; the pine 300 contained a total of 2.80 ± 0.18 mg DOC/g biochar, the pine 400 contained 1.96 ± 0.31 mg DOC/g biochar and the pine 500 contained 1.40 ± 0.19 mg DOC/g biochar (Fig. 1). The lower temperature biochars with a more acidic pH (Additional file 1: Fig. S2) may be more water soluble, which may explain the release of more water soluble fragments (i.e., DOC) than the higher temperature biochars that exhibit more hydrophobic characteristics (Xiao et al. 2014).

The ozone treated biochars had significantly more DOC in their filtrate. The wet ozonized P300 had six times more DOC materials compared to the non-ozonized P300 (Fig. 1). The wet ozonized P400 released the highest DOC amount among the pine biochars at 23.49 ± 1.70 mg DOC/g biochar, which was twelve times more DOC than the non-ozonized pine 400 (Fig. 1). Wet-ozone treatment of P500 also generated some DOC, but only increased by a factor of 2 to 3. The dry-ozonized rogue biochar released the most DOC material (34.49 ± 1.00 mg DOC/g biochar). The effect of the generated DOC materials was tested on the growth of *Pseudomonas putida* and cyanobacteria *Synechococcus elongatus* PCC 7942 as follows.

### Toxicity assay: effect of DOC of biochar filtrates on *P. putida*

The filtrates from pine 400 biochar and rogue biochar were used for the toxicity assay as they generated the highest amount of DOC materials upon ozone treatment. In addition, the pine 400 wet ozonized biochar showed great efficiency at solubilizing phosphate from hydroxyapatite material (Sacko et al. 2020) and the dry ozonized rogue biochar showed a high cation exchange capacity (Kharel et al. 2019). Before these biochars can be considered for application into the soil, it is important to know how they would affect soil microbes.

**Fig. 2:**
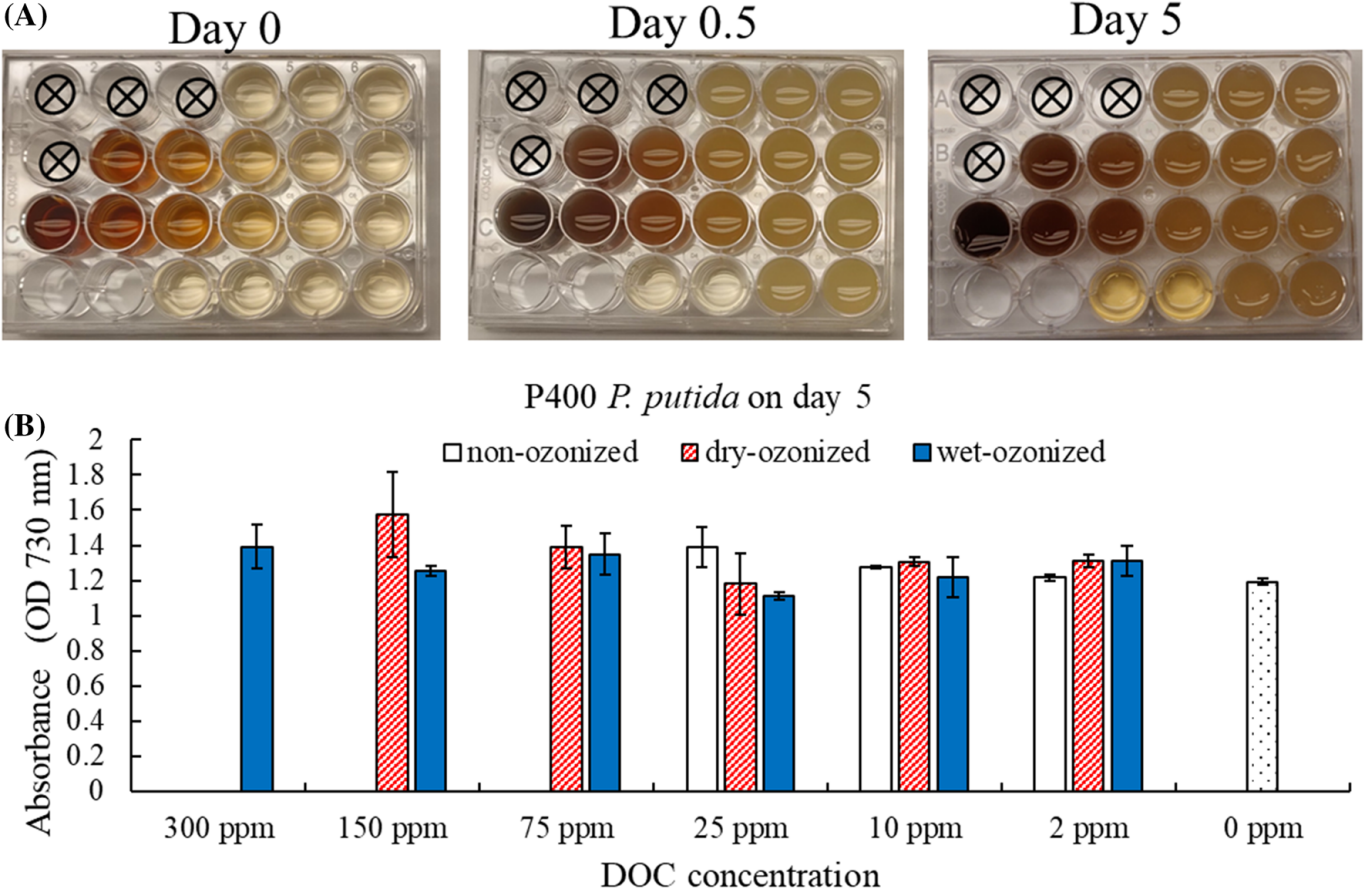
Growth assay of *P. putida* in incubation with filtrates from wet-ozonized, dry-ozonized, and non-ozonized pine 400 biochar at different DOC concentrations. **A** Photographs of multi-well plate with P400 biochar filtrates inoculated with *P. putida* on day 0, day 0.5 and day 5 of the growth assay. The photographs shown are one of the two replicates. The setup of the plate is shown in Table 1. **B** Optical density (OD730) of *P. putida* liquid culture after 5 days of incubation with P400 biochar filtrates. The error bars on the graph represent the standard deviation of the 2 multi-well plates (*n* = 2)

The growth assay of *P. putida* incubation with the P400 biochar filtrate showed that the *P. putida* growth was not inhibited at any of the tested DOC concentrations of ozonized P400 filtrate and non-ozonized P400 filtrate (Fig. 2). The photograph of the multi-well plates of the *P. putida* incubation with the P400 filtrates showed that there was cell growth, as seen by the turbidity of the liquid cells in each well (Fig. 2A). The optical density (OD730) measurements of the cells supported these observations (Fig. 2B). Note that the OD730 data reported were all calculated by subtracting the scattering caused by the biochar filtrates and other particles. That is the OD730 reported is only reflecting the growth of the cells. The OD730 from the *P. putida* incubated with the filtrates from P400 (non-ozonized, wet-ozonized, and dry-ozonized) at DOC concentrations 2–25 ppm were somewhat similar to that of the 0-ppm control. The *P. putida* incubation with the filtrates from the wet-ozonized pine 400 at high DOC concentrations also had comparable OD730 measurements to that of the 0-ppm “no DOC” control; no statistical difference was observed (Fig. 2B). The filtrate from the non-ozonized P400 did not have enough DOC, therefore, that assay was limited to low DOC concentrations. The OD730 of the *P. putida* growth was also recorded daily for up to 5 days when incubated with non-ozonized, wet-ozonized, and dry-ozonized pine 400 biochar filtrates. The data showed that even at high DOC concentrations (150 ppm, 300 ppm), the growth rate of *P. putida* was not inhibited (Additional file 1: Figs. S3, S4, S5).

**Fig. 3:**
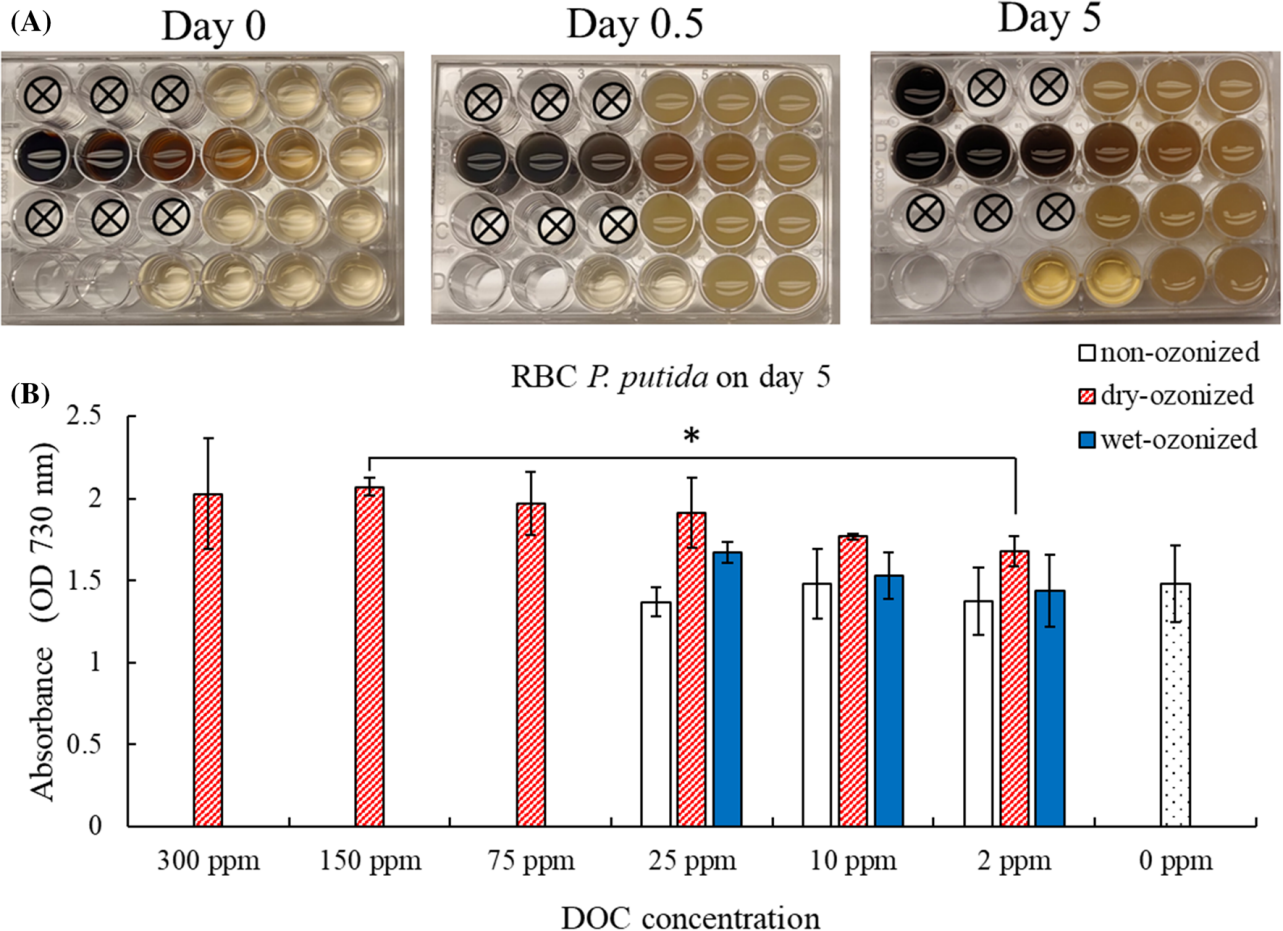
Growth assay of *P. putida* in incubation with filtrates from wet-ozonized, dry-ozonized, and non-ozonized rogue biochar at different DOC concentrations. **A** Photographs of multi-well plate with RBC biochar filtrates inoculated with *P. putida* on day 0, day 0.5 and day 5 of the growth assay. The photographs shown are one of the two replicates. The setup of the plate is shown in Table 1. **B** Optical density (OD730) of *P. putida* after 5 days of incubation with RBC biochar filtrates. The error bars on the graph represent the standard deviation of the 2 multi-well plates (*n* = 2). At day 5, the *P. putida* inoculated in the well with 300 ppm of the dry-ozonized RBC filtrate had too much growth and OD730 could not be measured; therefore, the content of the well was split in two wells and their OD730 measurements were added. The asterisks brackets (*) show significant difference (*p* < 0.05) between treatments

The toxicity assay conducted with the filtrates from rogue biochar (RBC) also showed that *P. putida* grew at all the different DOC concentrations tested (Fig. 3A). The optical density measurements confirmed these observations (Fig. 3B). The non-ozonized rogue biochar filtrate and the wet-ozonized rogue biochar filtrate did not have any major effect on the growth rate of *P. putida* at the DOC concentrations tested (Additional file 1: Figs. S6, S7). The *P. putida* incubation with the filtrate from the dry-ozonized rogue biochar at DOC concentrations of 2 ppm and 10 ppm had similar growth to that of the non-ozonized and wet-ozonized rogue biochar as shown by the OD730 data on day 5 (Fig. 3B). At higher DOC concentrations from the dry-ozonized rogue biochar, the growth of *P. putida* appeared to be stimulated in comparison with that at lower DOC concentrations. The *P. putida* cells culture density growth measured as OD730 in the medium with 150 ppm DOC was higher than that with 2 ppm DOC (statistical *p* value: 0.018). However, when compared to the control, there was no statistical difference. The *P. putida* cells culture of the 0 ppm DOC (“no DOC” control) had an OD730 of 1.48 ± 0.23 which was slightly smaller (statistic *p* value: 0.056) than that of the 150 ppm DOC with a measured OD730 of 2.07 ± 0.05. The 300 ppm DOC *P. putida* cell culture also showed a slightly higher OD730 (2.03 ± 0.34) compared to the no DOC control (statistic *p* value 0.1071) (Fig. 3B). The cell culture density growth recorded daily as OD730 also showed that *P. putida* grew slightly better with the dry-ozonized RBC filtrate at high DOC concentrations compared to lower DOC concentrations (Additional file 1: Fig. S8).

Low temperature-pyrolysis-produced biochars such as P400 have greater amounts of microbial inhibitory compounds, such as polycyclic aromatic hydrocarbons (PAH’s), furans, volatile organic compounds, etc. (Lyu et al. 2016; Smith et al. 2016). We expected the growth of *P. putida* to be inhibited by the filtrate from P400, but that was not the case. This may be because *P. putida* is capable of degrading several aromatic compounds that may be toxic to other microorganisms (Nogales et al. 2017).

Overall, the growth of the soil bacterium *P. putida* was not inhibited by the filtrates from the wet-ozonized pine 400 and the dry-ozonized rogue biochar. At high DOC concentrations (300 ppm), these filtrates may have some slight stimulatory effect to the growth of *P. putida,* which may be due to *P. putida's* ability to degrade and possibly utilize several aromatic compounds (Nogales et al. 2017).

### Toxicity assay: effect of DOC of biochar filtrates on cyanobacteria *Synechococcus elongatus* PCC 7942

**Fig. 4:**
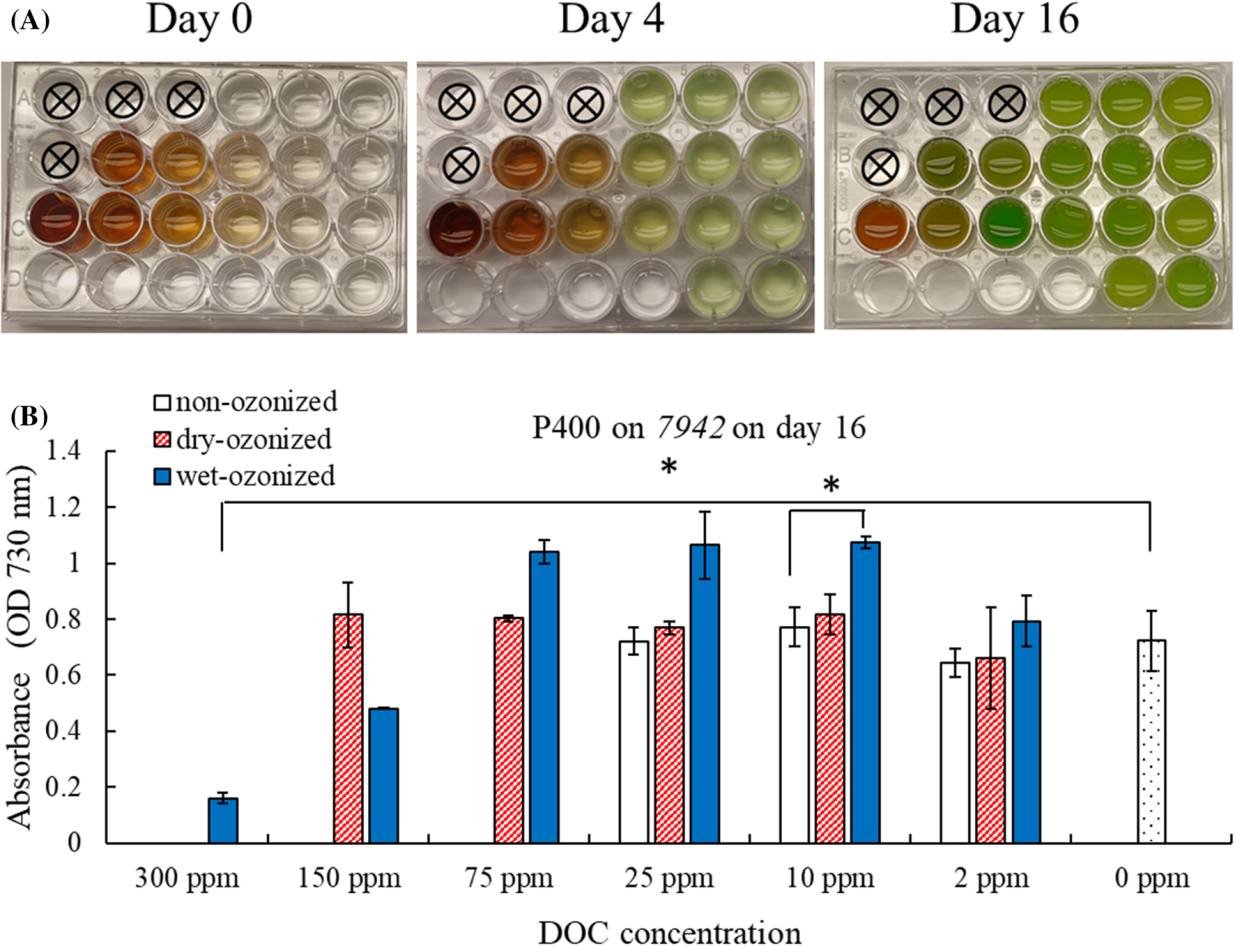
Growth assay of *Synechococcus elongatus* PCC 7942 (7942) in incubation with filtrates from wet-ozonized, dry-ozonized, and non-ozonized pine 400 (P400) biochar at different DOC concentrations. **A** Photographs of multi-well plate with P400 biochar filtrates inoculated with 7942 on day 0, day 4 and day 16 of the growth assay. The photographs shown are one of the two replicates. The setup of the plate is shown in Table 1. **B** Optical density (OD730) of 7942 after 16 days of incubation with P400 biochar filtrates. The error bars on the graph represent the standard deviation of the 2 multi-well plates (*n* = 2). The asterisks brackets (*) show significant difference (*p* < 0.05) between treatments

The effect of ozonized biochar water extractable organic carbon was tested on the growth of cyanobacteria *Synechococcus elongatus* PCC 7942 (7942). The 7942 was able to grow when incubated with filtrates from non-ozonized, wet-ozonized, and dry-ozonized pine 400 (Fig. 4). At a DOC concentration of 25 ppm, the filtrate from the non-ozonized P400 (OD730 0.72 ± 0.05) and dry-ozonized P400 (OD730 0.77 ± 0.02) had no major effect on the growth of 7942 as their OD730 was similar to that of the 0-ppm control (OD730 0.72 ± 0.11) as recorded on day 16 (Fig. 4). The 25-ppm DOC from the wet-ozonized P400, on the other hand, slightly stimulated the growth of 7942 with an OD730 of 1.06 ± 0.12 on day 16, which was slightly greater than the 0-ppm “no DOC” control (statistic *p* value: 0.160). The wet-ozonized P400 filtrate stimulated the growth of 7942 also at 75 ppm DOC (statistic *p* value: 0.098). However, at higher DOC concentrations (150 ppm and 300 ppm), the growth of 7942 was significantly inhibited (Fig. 4).

The DOC extracted from the dry-ozonized P400 biochar had no major effect on the growth of 7942 at the concentrations tested (2–150 ppm DOC) (Fig. 4). The growth rate was also recorded and showed that the P400 non-ozonized biochar filtrate inhibited the growth rate of 7942: from day 10 to day 14, the OD730 of 7942 incubated with non-ozonized P400 filtrate (25 ppm) was slightly less than that of the control 0 ppm (Additional file 1: Fig. S9). It is not until day 16 that the OD730 of the 7942 incubated with non-ozonized P400 filtrate (25 ppm) caught up to the OD730 of the 7942 incubated with the control at 0 ppm DOC (no DOC) (Additional file 1: Fig. S9). The 7942 incubated with the filtrate from the dry-ozonized pine 400 at 25 ppm DOC grew at the same rate as the 0-ppm control (Additional file 1: Fig. S10). 7942 incubated with the wet-ozonized P400 filtrate at 25 ppm also grew at the same rate as the 0 ppm up until day 10, then grew slightly better than the control (Additional file 1: Fig. S11). Also as shown in Fig. 4, at a DOC concentration of 10 ppm, the 7942 grew significantly better in presence of the wet-ozonized P400 DOC compared to the non-ozonized P400 DOC (statistic *p* value: 0.027). These observations indicated that at 10 ppm and possibly 25 ppm DOC, the non-ozonized P400 filtrate had a slight inhibition on the growth of 7942 compared to the dry and wet-ozonized P400 filtrate. It is possible that:

1. Some inhibitory compounds generally present in pine 400 biochar (Smith et al. 2016) may have been reduced upon ozonization of biochar;
2. Ozonization of biochar have caused the release of more nutrients which may have benefited the growth of 7942.

**Fig. 5:**
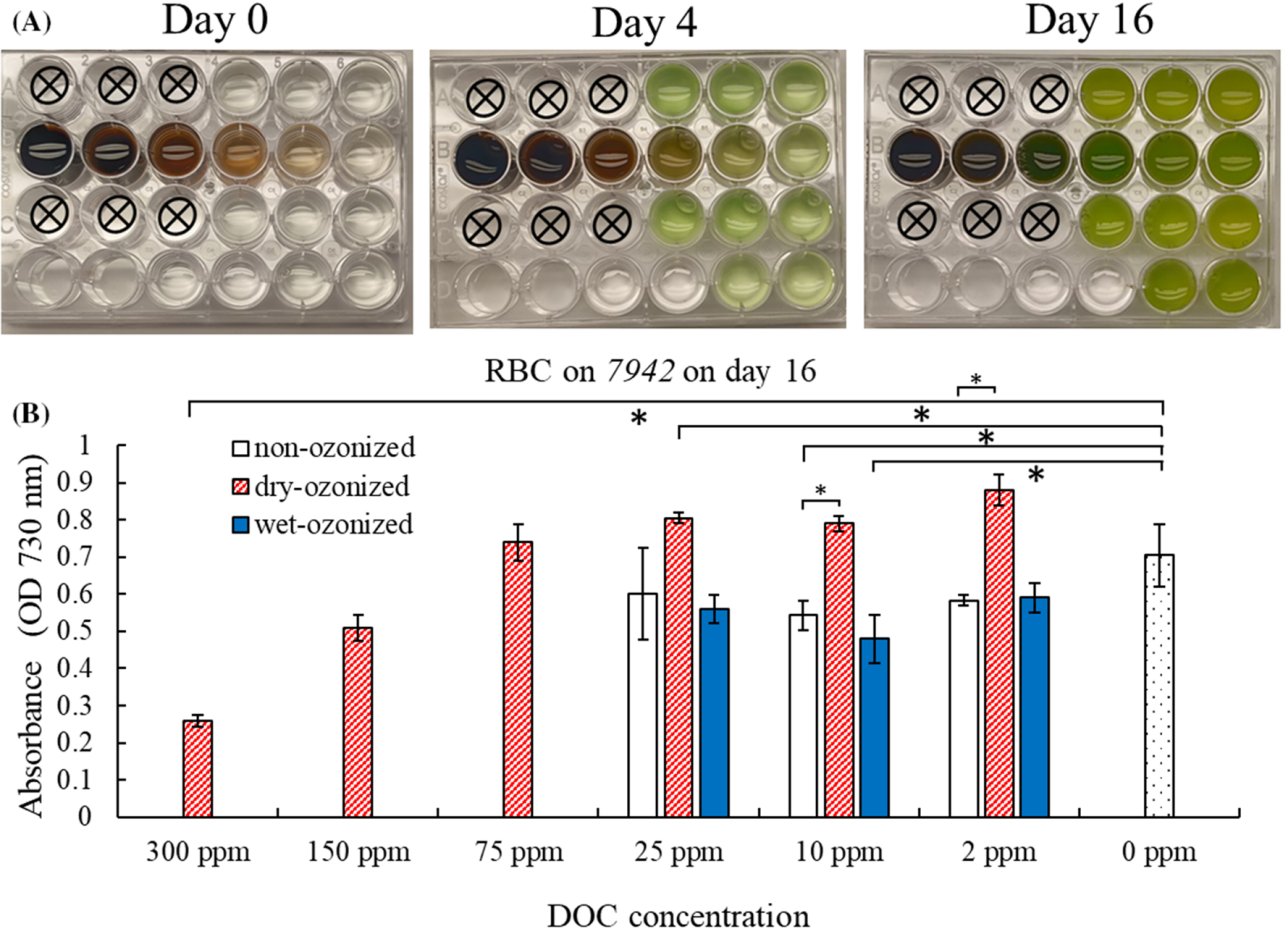
Growth assay of *Synechococcus elongatus* PCC 7942 (7942) in incubation with filtrates from wet-ozonized, dry-ozonized, and non-ozonized rogue biochar (RBC) at different DOC concentrations. **A** Photographs of multi-well plate with RBC biochar filtrates inoculated with 7942 on day 0, day 4 and day 16 of the growth assay. The photographs shown are one of the two replicates. The setup of the plate is shown in Table 1. **B** Optical density (OD730) of 7942 after 16 days of incubation with RBC filtrates. The error bars on the graph represent the standard deviation of the 2 multi-well plates (*n* = 2). The asterisks brackets (*) show significant difference (*p* < 0.05) between treatments

The effect of the DOC extracted from the rogue biochar was also tested. On day 16, the PCC 7942 incubated with the dry-ozonized rogue biochar filtrate at 2–25 ppm had higher OD730 reading (growth) than when 7942 was incubated with the filtrates from the wet-ozonized RBC at 2–25 ppm and from the non-ozonized RBC at 2–25 ppm (Fig. 5). In addition, at DOC concentrations of 2–10 ppm, the non-ozonized RBC filtrates significantly inhibited the growth of 7942 compared to the “no DOC” control (statistical *p* value < 0.05). This indicates that at these DOC concentrations, the cells grow better when incubated with the dry-ozonized rogue biochar filtrate than with the non-ozonized rogue biochar filtrate. It is possible that dry-ozonization reduces the presence of inhibitory compounds that may be present in RBC. It is also possible that dry-ozonization caused the release of more nutrients in the filtrate of RBC. However, at higher concentrations (300 ppm), the dry-ozonized RBC filtrate significantly inhibited the growth of 7942 compared to the 0-ppm control (statistic *p* value: 0.013). Furthermore, we observed that the growth rate of 7942 was slowed in the presence of non-ozonized rogue biochar filtrate (at 2–25 ppm DOC) until day 14 (Additional file 1: Fig. S12). The growth rate of 7942 was also slowed when incubated with the dry-ozonized RBC filtrate at 75 ppm for 12 days, then picked back up on days 14–16 (Additional file 1: Fig. S13). At high DOC concentrations (150–300 ppm), the dry-ozonized RBC filtrate significantly inhibited the growth rate of 7942, which was still growing, but at a much slower rate than when it was incubated with the control 0 ppm DOC (Additional file 1: Fig. S13). The filtrate from the wet-ozonized RBC had a similar effect on the growth pattern of 7942 as the non-ozonized RBC; at 2–25 ppm DOC, they had a slower growth than the 0-ppm control for the first 14 days (Additional file 1: Fig. S14).

The filtrate from the dry-ozonized rogue biochar at 300 ppm DOC was much less inhibitory to the growth of PCC 7942 than the filtrate from wet-ozonized P400 biochar at 300 ppm DOC (Figs. 4, 5, Additional file 1: Figs. S11, S13). The rogue biochar being made at higher temperatures (700 °C) may have less inhibitory compounds to start with, compared to the pine 400 made at lower temperatures (400 °C). The filtrate from dry ozonized rogue biochar at low DOC concentrations (2–25 ppm) appeared to slightly stimulate the growth of cyanobacteria PCC 7942, and have no inhibitory effect on *P. putida* at high concentrations. In addition, the dry-ozonized rogue biochar was recently characterized by our lab to be able retain and exchange cations 7–9 time more than the non-ozonized control (Kharel et al. 2019). While the results presented here are empirical evidence that the ozonization of biochar reduces its inhibitory effect on the growth of *P. putida* KT2440 and *S. elongatus* PCC 7942, to the best of our knowledge this is the first study that investigated the effect of ozonized biochar filtrates on microorganisms. These results are of great importance as it showed a potential reduction of inhibition upon ozonization; these findings call for more investigation into the property and effect of ozonized biochar on a wider range of microorganism for its use as a soil amendment.

### Determination of the anions released by biochar using ion chromatography

In the literature, studies have shown that some biochars may contain nutrients, such as nitrate, phosphorus, ammonium that are released over time when the biochar is introduced into the soil (Mukherjee and Zimmerman 2013; Glaser and Lehr 2019). Earlier, we hypothesized that the stimulatory effect seen in the incubation of the bacteria with the ozonized biochar filtrates may be owing to some nutrients that are released following ozonization. In this section, we tested the presence of several anions in the filtrate of biochar before and after ozonization.

We found that the filtrate from the dry-ozonized rogue biochar contained larger amounts of phosphate (32.80 mg/L ± 0.26) and nitrate (22.75 mg/L ± 0.20), compared to the filtrate from the wet-ozonized pine 400, where the levels were undetectable (Table 2). The release of nitrate and phosphate from the dry-ozonized rogue biochar were certainly caused by ozone treatment, given that the non-ozonized RBC only contained a small amount of phosphate (4.92 mg/L ± 0.60) and an undetectable amount of nitrate (Table 2). Ozone treatment of rogue biochar made it more polar and water soluble, as shown by the acidic pH of the ozonized biochar in Additional file 1: Fig. S2. This may have facilitated the release of nutrients. It is possible that the extra phosphate in the filtrate of the dry-ozonized RBC favored the growth of the *P. putida* and *S. elongatus* PCC 7942 (at low DOC concentration) compared to the non-ozonized RBC. Nitrate and phosphate are important nutrients in the soil; another potential benefit of ozonized rogue biochar would be to deliver more of these nutrients in the soils with low initial amounts of available phosphorus.

**Table 2:** Measurements of phosphate, chloride, nitrate, and sulfate concentrations in the filtrate of the non-ozonized pine 400 (P400 UN), wet-ozonized pine 400 (P400 90 W), non-ozonized rogue biochar (RBC UN) and dry-ozonized rogue biochar (RBC 90D)

	Phosphate (mg/L)	Chloride (mg/L)	Nitrate (mg/L)	Sulfate (mg/L)
P400 UN	0	0.05 ± 0.03	0	0
P400 90W	0	0.10 ± 0.03	0	0
RBC UN	4.92 ± 0.60	12.76 ± 0.07	0	27.60 ± 0.56
RBC 90D	32.80 ± 0.26	4.22 ± 0.32	22.75 ± 0.20	22.92 ± 0.76

The filtrate was obtained from the 2nd wash (25 mL) of 1.5 g biochar. The values shown are averages of 3 replicates (*n* = 3). The conductivity signals by IC are shown in Additional file 1: Fig. S15

Another observation made by the ion chromatography (IC) analysis was that dry ozone treatment of rogue biochar significantly decreased the amount of chloride in the filtrate (Table 2). The IC conductivity of the filtrates is shown in Additional file 1: Fig. S15. Wet-ozonized P400 filtrate showed the presence of two compounds (IC elution peaks “a” and “b”) that were not present in the non-ozonized pine 400. The exact identities of these compounds are unknown, but we suspect them to be small carboxylated molecules produced by the cleavage of biochar olefinic groups by ozone. Literature showed that under certain conditions ozone can degrade humic acids into small organic acids, such as formic acid, oxalic acid, acetic acid, etc. (Kusakabe et al. 1990; Takahashi et al. 1995). Here, we suspect that wet ozonization of pine 400, generated some amount of these organic acids; signal “a” could be monocarboxylated organic anion, such as acetate (Scientific 2013); this was later supported as it has similar retention time with standard acetic acid (Additional file 1: Fig. S16).

Another hypothesis made earlier was that ozonization may reduce the amount of inhibitory compounds. Therefore, we also analyzed for the presence of some potential inhibitory compounds in the filtrates from biochar materials before and after ozonization.

### Identification of bacterial inhibitory compounds by GC–MS

The filtrates extracted from the non-ozonized P400, wet-ozonized P400, non-ozonized RBC, and dry-ozonized RBC were analyzed by GC–MS to see if they exhibited the presence of potential inhibitory compounds. Smith et al. (2013) found that the toxicity of biochar water soluble organic compounds (WSOC) on algal growth was mainly caused by the presence of certain aromatic species with negatively charged groups and that these inhibitory compounds are likely to contain a carboxyl group; these toxic compounds were particularly prevalent in pinewood-derived biochar and could originate from the degradation of lignin (Smith et al. 2013). Here, para-toluic acid and terephthalic acid were the major differentiating compounds that were detected. The summary of their detection among treatments is shown in Table 3. *p*-toluic acid was detected in non-ozonized pine 400 biochar filtrate but was not detected in wet-ozonized P400; *p*-toluic acid was not detected in non-ozonized rogue biochar and dry-ozonized rogue biochar filtrate (Table 3). In addition, terephthalic acid was also detected in non-ozonized P400 filtrate but was only present in trace amounts in wet-ozonized P400 filtrate. It is possible that the effect of ozonization degraded those potential inhibitory compounds (Chandrasekara Pillai et al. 2009; Zang et al. 2009), which in turn may have helped reduce the inhibitory effect of pine 400 filtrate. In addition, ozone has been used to degrade PAH’s (O’Mahony et al. 2006). The study here tested only two microorganisms. It is possible that these ozonized filtrates may have a different effect on other microorganisms with different metabolic pathways. Nonetheless, the observations are important; the decreased inhibitory effect of the ozonized biochar filtrate at the tested DOC concentrations further supports the potential application of ozonized biochar. An interesting future study would be to see if *P. putida* or other non-photosynthetic bacteria can utilize the DOC from the ozonized biochar when the latter is the only source of carbon in minimal media.

**Table 3:** Detection of potential bacterial inhibitory compounds (*p*-toluic acid and terephthalic acid) in the filtrate of non-ozonized pine 400 (P400 UN), wet-ozonized pine 400 (P400 UN), non-ozonized rogue biochar (RBC UN) and dry-ozonized rogue biochar (RBC 90D)

	*p*-toluic acid	terephthalic acid
P400 UN	(+++) detected	(+++) detected
P400 90W	(−) not detected	(+) trace amount detected
RBC UN	(−) not detected	(−) not detected
RBC 90W	(−) not detected	(−) not detected

The GC–MS spectra of the samples are shown in Additional file 1: Figs. S17 and S18

## Conclusion

We previously showed that ozonization can significantly improve the CEC of biochar (Kharel et al. 2019). A high CEC biochar would have a greater nutrient retention capability, which is an important feature for its use as a soil amendment. Here, we conducted a toxicity assay of the filtrates from ozonized biochars on a soil bacterium (*P. putida*) and a freshwater bacterium (*S. elongatus* PCC 7942). We found that the water-soluble organic compounds from the ozone treated pine 400 biochar did not have any inhibitory effect on *P. putida*. Similarly, the filtrate from the high CEC rogue biochar did not show any inhibitory effect on *P. putida*. On the contrary, *P. putida* slightly grew better with the filtrate from the dry-ozonized rogue biochar at high DOC concentrations.

The toxicity assay performed on the freshwater cyanobacteria *S. elongatus* PCC 7942 had different results. The wet-ozonized P400 filtrate and the dry-ozonized RBC filtrate at low DOC concentrations (10–75 ppm) slightly improved the growth of 7942; but at higher DOC concentration (150–300 ppm) they inhibited the growth of 7942. In addition, we found that at a similar DOC concentration of 25 ppm, the non-ozonized P400 filtrate inhibited the growth rate of PCC 7942, but the wet-ozonized P400 filtrate did not. Furthermore, we found the presence of potential inhibitory compounds *p*-toluic acid and terephthalic acid in non-ozonized P400 filtrate, but only trace amounts in wet-ozonized P400 filtrate. Ozonization may have degraded these potential inhibitory compounds. Finally, we found that ozonization increased the release of phosphate and nitrate from rogue biochar, which may have provided extra nutrients for the growth of the bacteria. While many studies showed the efficiency of biochar in improving microbial activities via biochar-microbe interactions (Zhu et al. 2017), others have shown that in the short term, biochar pores may not be a preferred habitat for microbes (Quilliam et al. 2013). Given that the filtrate collected from ozonized biochar had no inhibitory effect on *P. putida*, a future and interesting study would be the use of ozonized biochar as an inoculum carrier and see how easily bacteria populate the ozonized biochar.

### Supplementary Information

**Additional file 1.** Ozonized biochar filtrate effects on the growth of *Pseudomonas putida* and cyanobacteria *Synechococcus elongatus* PCC 7942. Additional figures show the additional data on the growth assay (**Figure S1**) and optical density measurements (**Figure S3–S14**). Additional data on the biochars sources (**Figure S2**) is also shown. **Figure S15–S18** represent data on ion chromatography and the GC-MS spectra of the biochar filtrates.

## Data Availability

All data and materials are available as reported in the research article and its supporting information document that will be posted on the journal website.
