# Supplementary material for: Ozonized biochar filtrate effects on the growth of Pseudomonas putida and cyanobacteria Synechococcus elongatus PCC 7942
Source: Bioresour Bioprocess. 2022 Jan 6;9(1):2. doi: 10.1186/s40643-021-00491-2 (PMC10991886; doi:10.1186/s40643-021-00491-2)
Supplement: Supplementary file 1 — Additional file 1. Ozonized biochar filtrate effects on the growth of Pseudomonas putida and cyanobacteria Synechococcus elongatus PCC 7942. Additional figures show the additional data on the growth assay (Figure S1) and optical density measurements (Figure S3–S14). Additional data on the biochars sources (Figure S2) is also shown. Figure S15–S18 represent data on ion chromatography and the GC-MS spectra of the biochar filtrates. [file 40643_2021_491_MOESM1_ESM.pdf]

SUPPORTING INFORMATION

Ozonized biochar filtrate effects on the growth of *Pseudomonas putida* and cyanobacteria *Synechococcus elongatus* PCC 7942

Oumar Sacko [1], Nancy L. Engle [2], Timothy J. Tschaplinski [2], Sandeep Kumar [3], and James Weifu Lee [1*]

[1]Department of Chemistry and Biochemistry, Old Dominion University, Norfolk, VA 23529

[2]Oak Ridge National Laboratory, PO Box 2008, Oak Ridge, TN 37831

[3]Department of Civil and Environmental Engineering, Old Dominion University, Norfolk, VA 23529

*Corresponding Author Email: jwlee@odu.edu

Additional experimental data: 18 Figures (S1-S18) on pages S2-S13

The Figures are additional data on the growth assay (Figure S1) and optical density measurements (Figure S3-S14). Additional data on the biochars sources (Figure S2) is also shown. Figure S15-S18 represent data on ion chromatography and the GC-MS spectra of the biochar filtrates.

Key words: ozonized biochar filtrate, dissolved organic carbon, bioassay, biological effects of ozonized biochar substances

A

	1	2	3	4	5	6
A	UN 300ppm	UN 150ppm	UN 75ppm	UN 25ppm	UN 10ppm	UN 2ppm
B	90D 300ppm	90D 150ppm	90D 75ppm	90D 25ppm	90D 10ppm	90D 2ppm
C	90W 300ppm	90W 150ppm	90W 75ppm	90W 25ppm	90W 10ppm	90W 2ppm
D	empty	empty	empty	empty	empty	empty

B

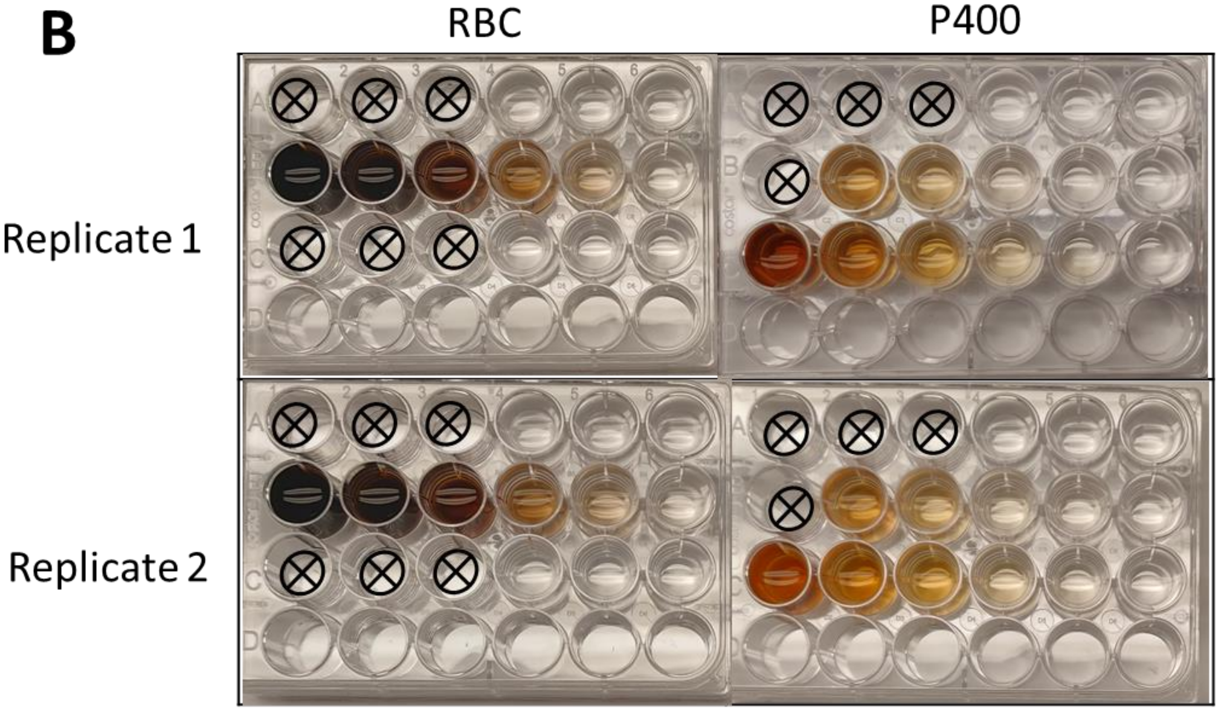

Figure S1: Photographs of the multi-well plates with the filtrates used as a control for the biochar filtrate toxicity assay. The layout of the multi-well plate is shown in A) the non-ozonized biochar filtrates, dry-ozonized biochar filtrates and wet-ozonized biochar filtrates were in row A, row B and row C, respectively. The DOC concentration ranges from 2 ppm to 300 ppm going from right to left. The photographs of the two multi-well plates are shown in B) for the filtrates from the rogue biochar (RBC) and pine 400 biochar (P400). Wells shown with the cross are the wells where the DOC extracted from the biochar was too low to reach the desired DOC concentration of the well.

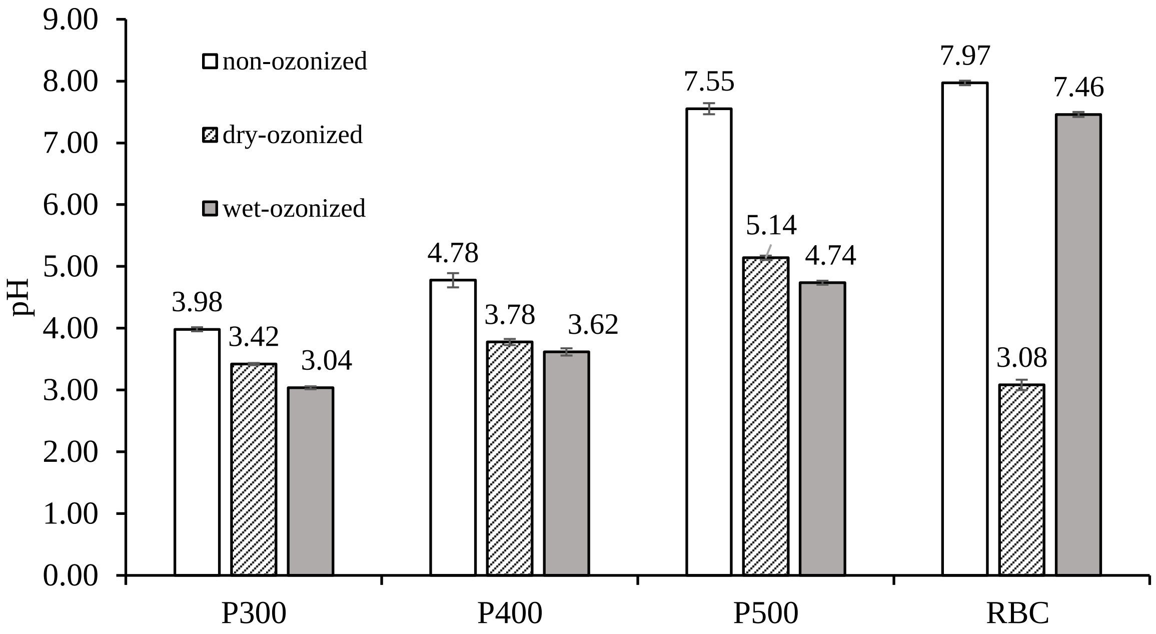

Figure S2: pH measurements of the pine 300 (P300), pine 400 (P400), pine 500 (P500) and rogue biochar (RBC) slurries. Error bars denote standard deviation of 3 replicates (n=3).

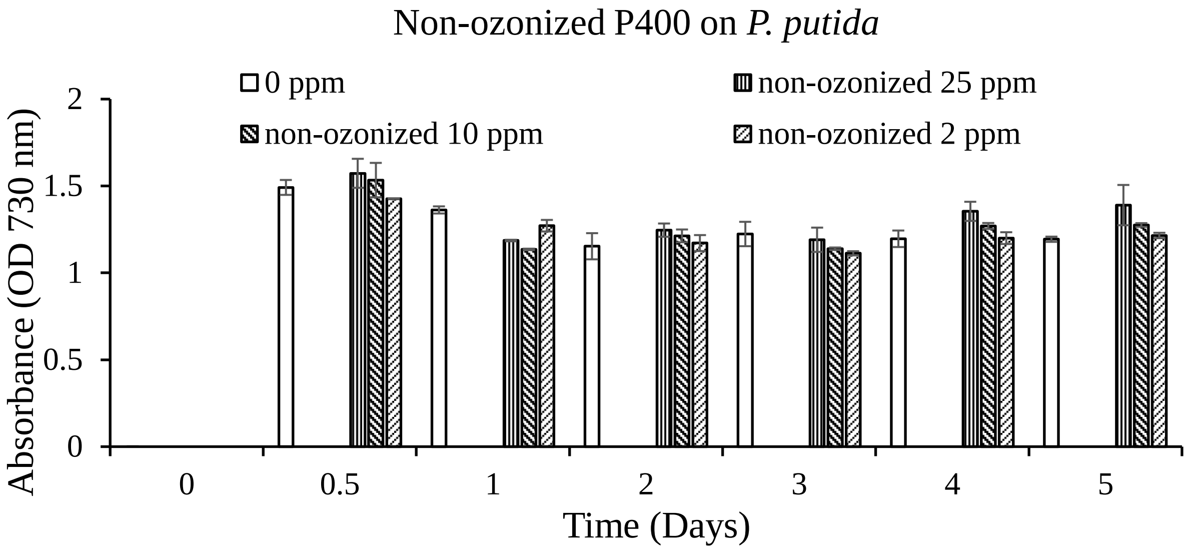

Figure S3: Optical density (OD730) of the growth of *P. putida* incubation with filtrates from non-ozonized pine 400 biochar at different DOC concentrations. The OD730 was measured every day for up to 5 days. The error bars on the graphs represent the standard deviation of the 2 multi-well plates (n=2).

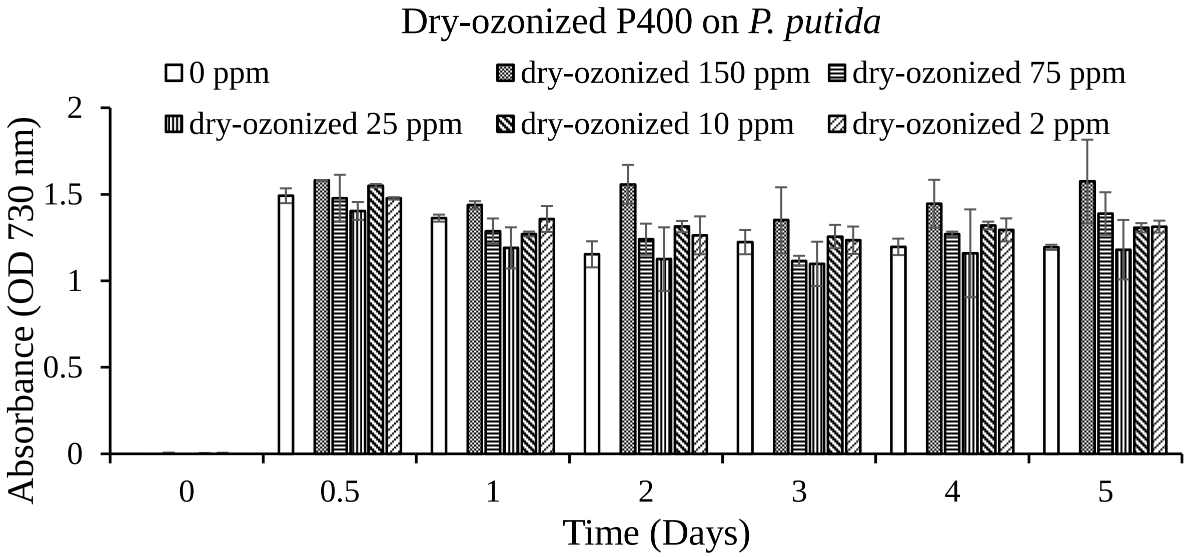

Figure S4: Optical density (OD730) of the growth of *P. putida* incubation with filtrates from dry-ozonized pine 400 biochar at different DOC concentrations. The OD730 was measured every day for up to 5 days. The error bars on the graphs represent the standard deviation of the 2 multi-well plates (n=2).

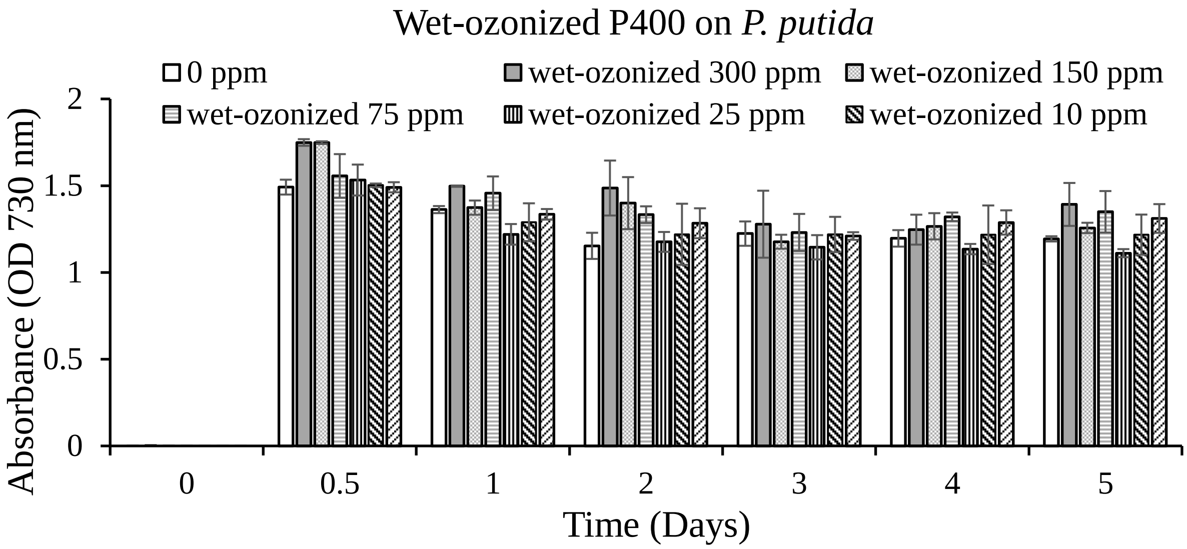

Figure S5: Optical density (OD730) of the growth of *P. putida* incubation with filtrates from wet-ozonized pine 400 biochar at different DOC concentrations. The OD730 was measured every day for up to 5 days. The error bars on the graphs represent the standard deviation of the 2 multi-well plates (n=2).

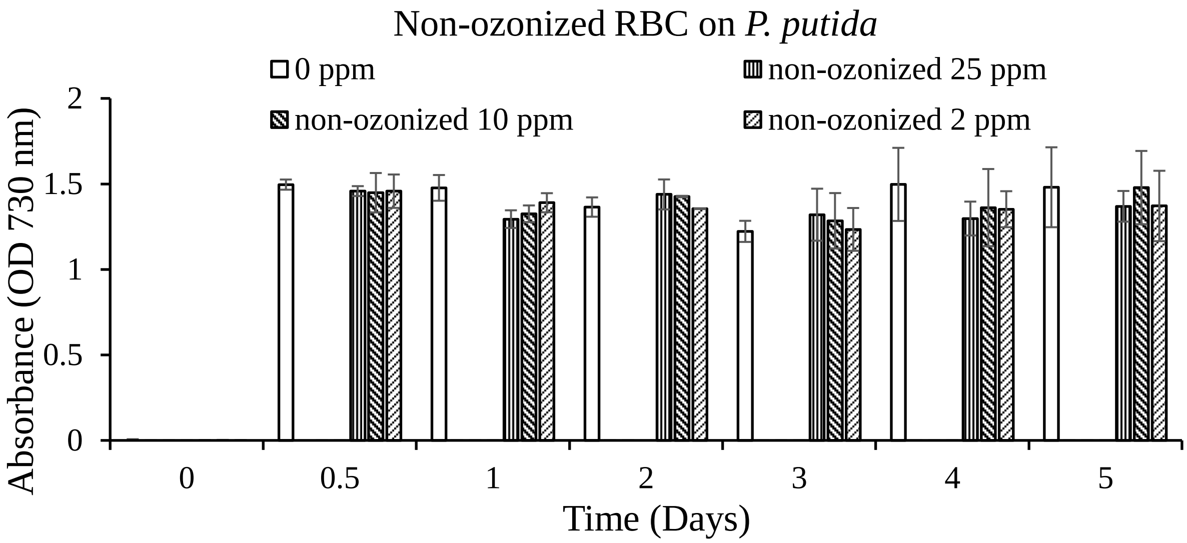

Figure S6: Optical density (OD730) of the growth of *P. putida* incubation with filtrates from non-ozonized rogue biochar (RBC) at different DOC concentrations. The OD730 was measured every day for up to 5 days. The error bars on the graphs represent the standard deviation of the 2 multi-well plates (n=2).

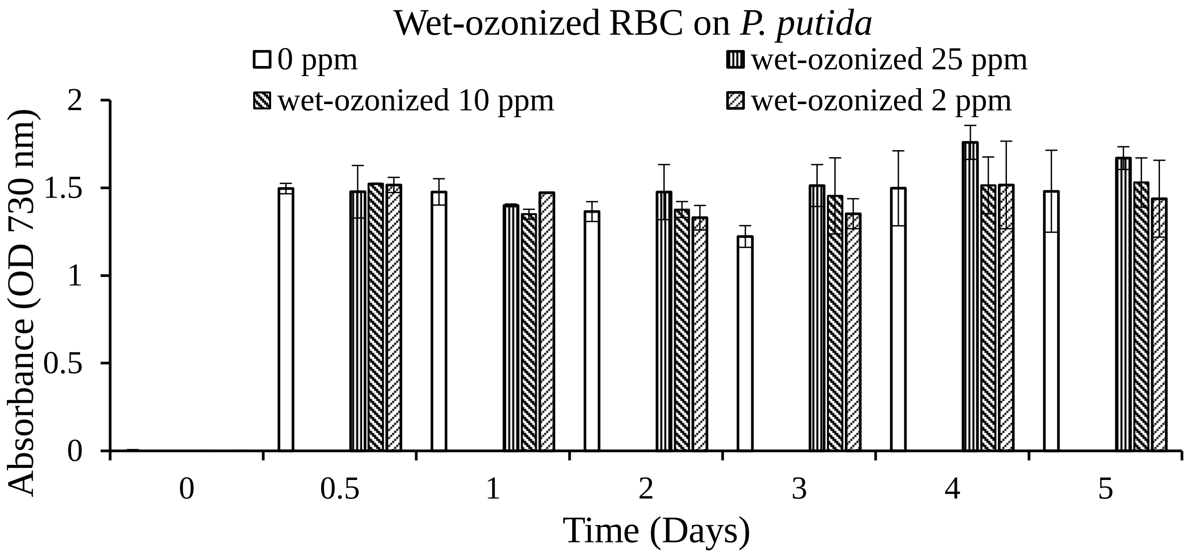

Figure S7: Optical density (OD730) of the growth of *P. putida* incubation with filtrates from wet-ozonized rogue biochar (RBC) at different DOC concentrations. The OD730 was measured every day for up to 5 days. The error bars on the graphs represent the standard deviation of the 2 multi-well plates (n=2).

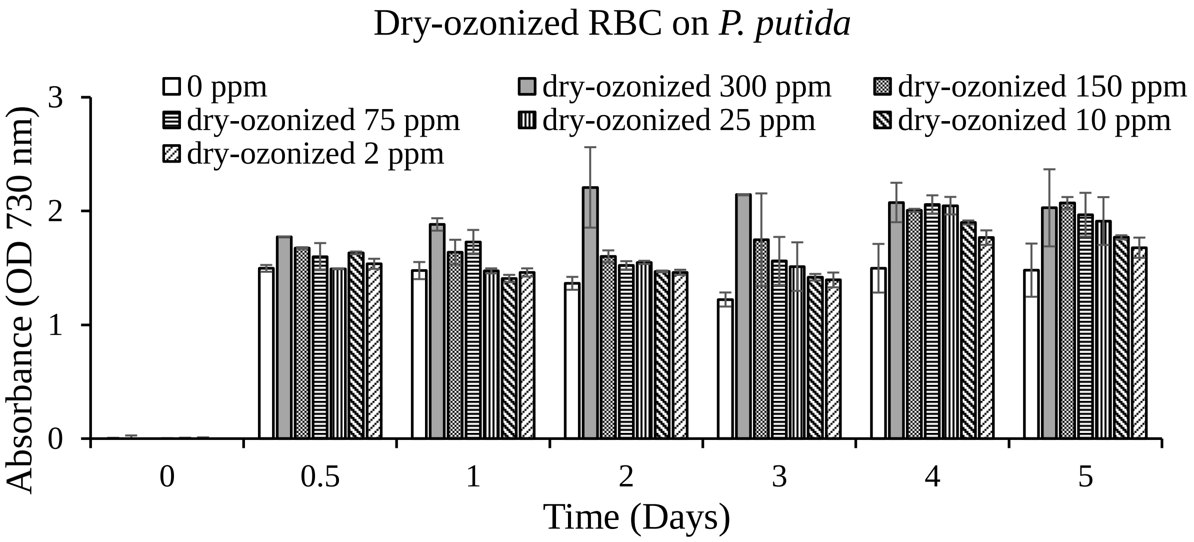

Figure S8: Optical density (OD730) of the growth of *P. putida* incubation with filtrates from dry-ozonized rogue biochar (RBC) at different DOC concentrations. The OD730 was measured every day for up to 5 days. The error bars on the graphs represent the standard deviation of the 2 multi-well plates (n=2).

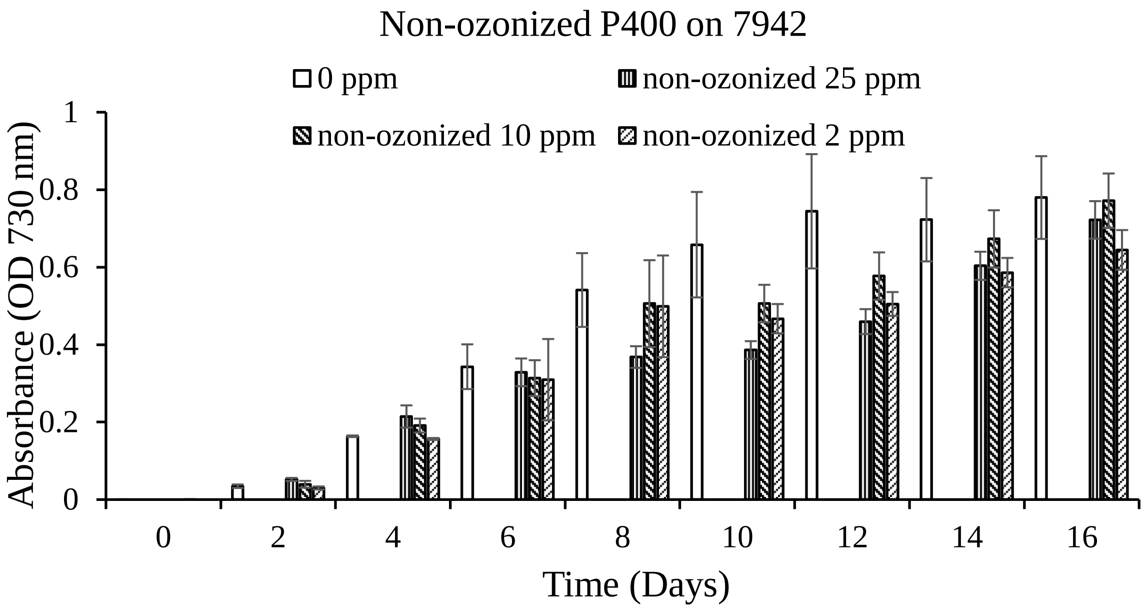

Figure S9: Optical density (OD730) of the growth of *synechococcus elongatus* PCC 7942 (7942) incubation with filtrates from non-ozonized pine 400 biochar at different DOC concentrations. The OD730 was measured every other day for up to 16 days. The error bars on the graphs represent the standard deviation of the 2 multi-well plates (n=2).

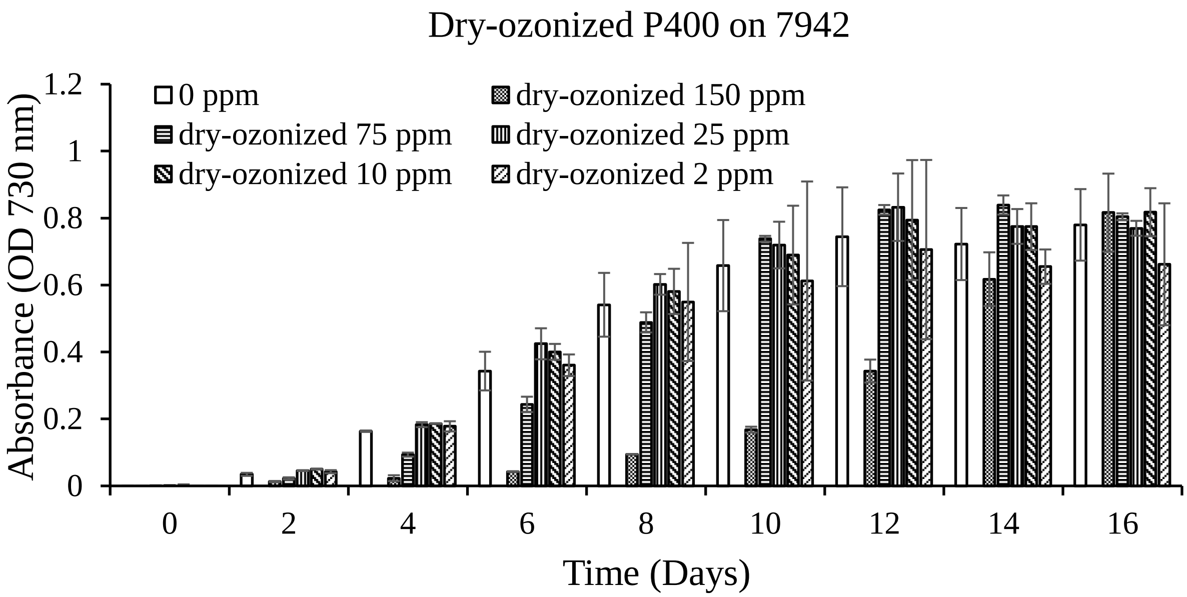

Figure S10: Optical density (OD730) of the growth of *synechococcus elongatus* PCC 7942 (7942) incubation with filtrates from dry-ozonized pine 400 biochar at different DOC concentrations. The OD730 was measured every other day for up to 16 days. The error bars on the graphs represent the standard deviation of the 2 multi-well plates (n=2).

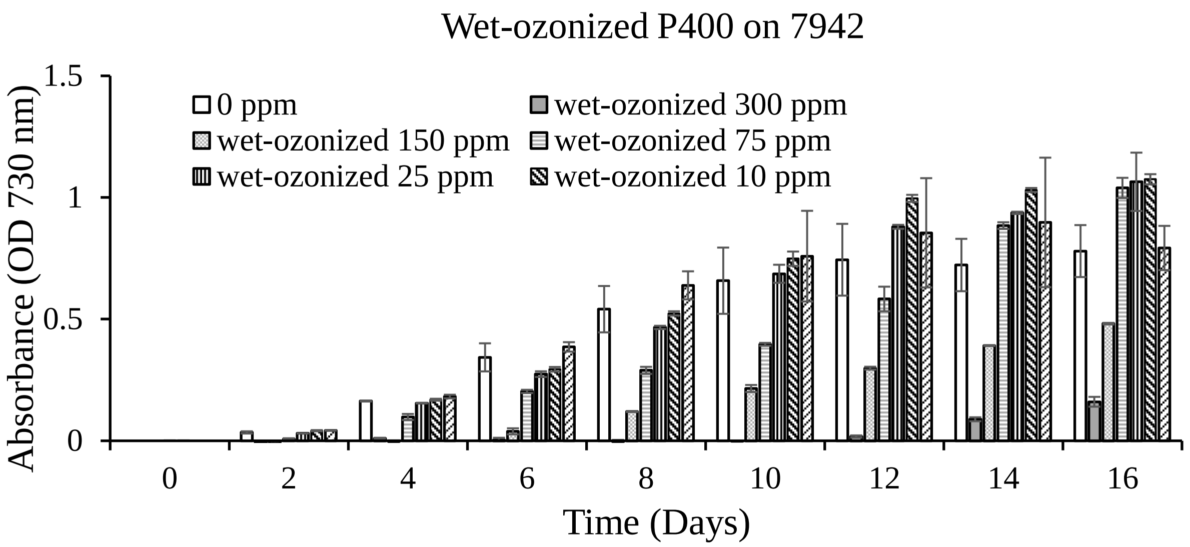

Figure S11: Optical density (OD730) of the growth of *synechococcus elongatus* PCC 7942 (7942) incubation with filtrates from wet-ozonized pine 400 biochar at different DOC concentrations. The OD730 was measured every other day for up to 16 days. The error bars on the graphs represent the standard deviation of the 2 multi-well plates (n=2).

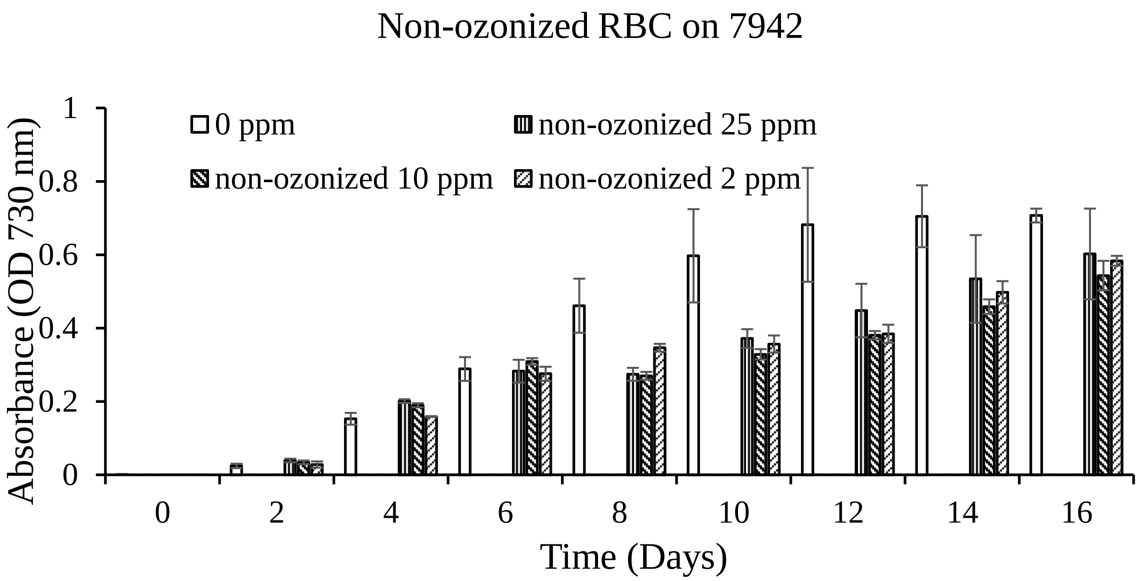

Figure S12: Optical density (OD730) of the growth of *synechococcus elongatus* PCC 7942 (7942) incubation with filtrates from non-ozonized rogue biochar (RBC) at different DOC concentrations. The OD730 was measured every other day for up to 16 days. The error bars on the graphs represent the standard deviation of the 2 multi-well plates (n=2).

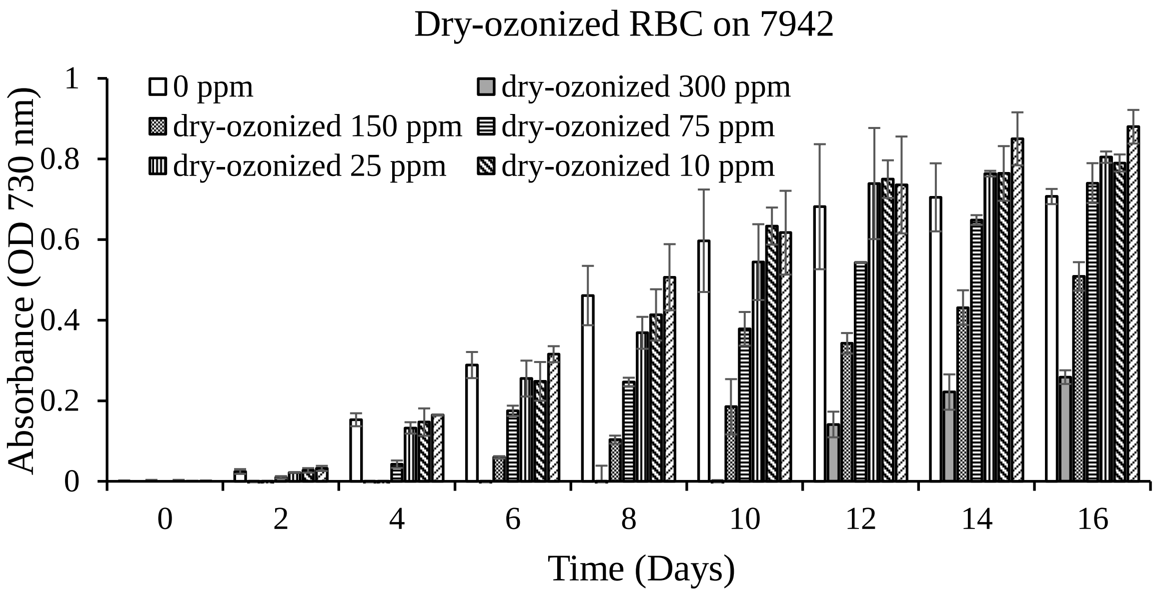

Figure S13: Optical density (OD730) of the growth of *synechococcus elongatus* PCC 7942 (7942) incubation with filtrates from dry-ozonized rogue biochar (RBC) at different DOC concentrations. The OD730 was measured every other day for up to 16 days. The error bars on the graphs represent the standard deviation of the 2 multi-well plates (n=2).

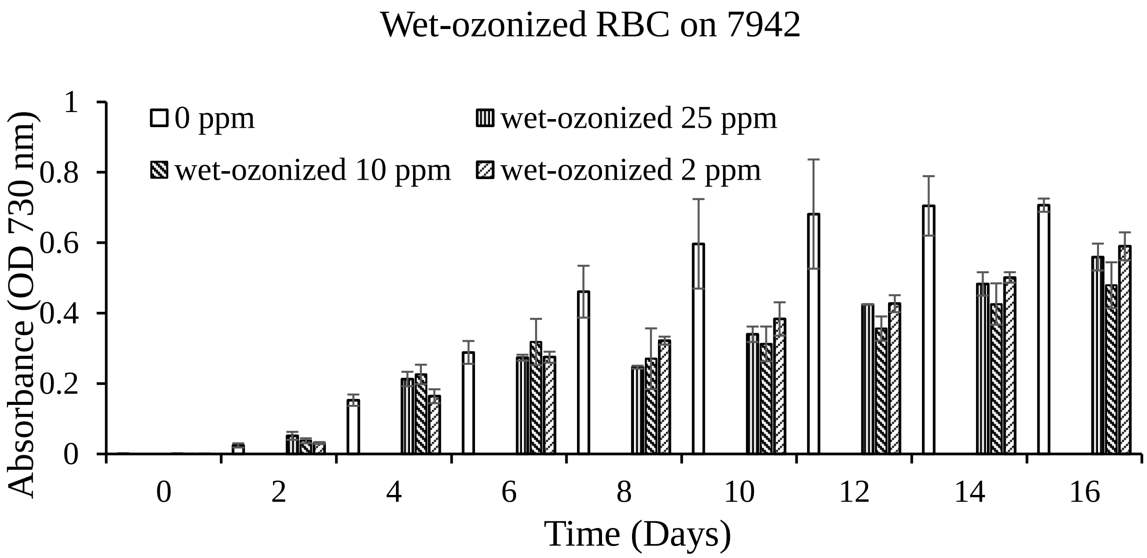

Figure S14: Optical density (OD730) of the growth of *synechococcus elongatus* PCC 7942 (7942) incubation with filtrates from wet-ozonized rogue biochar (RBC) at different DOC concentrations. The OD730 was measured every other day for up to 16 days. The error bars on the graphs represent the standard deviation of the 2 multi-well plates (n=2).

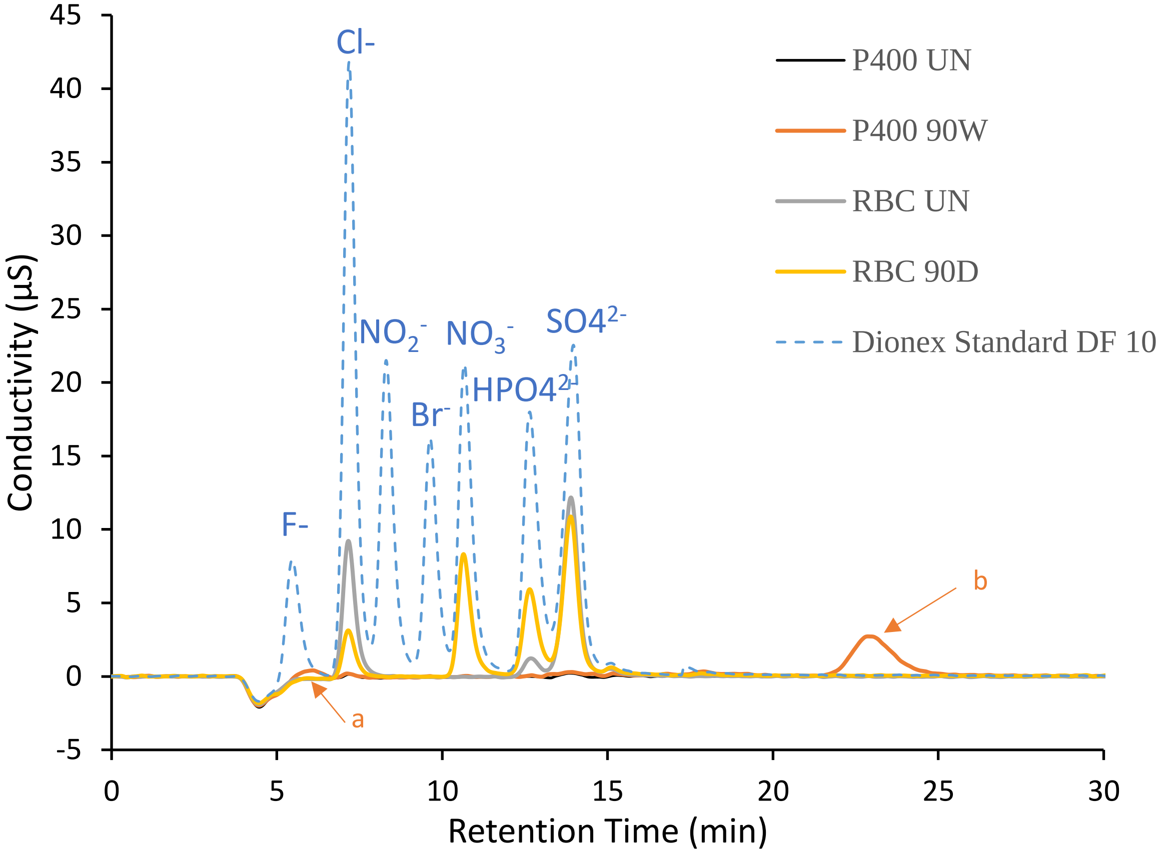

Figure S15: Conductivity signals of anions from the filtrate of the non-ozonized pine 400 (P400 UN) biochar, wet-ozonized pine 400 (P400 90W) biochar, non-ozonized rogue biochar (RBC UN) and dry-ozonized rogue biochar (RBC 90D). The standard Dionex seven (fluoride, chloride, nitrite, bromide, nitrate, phosphate, and sulfate) signals are also shown as a reference. The signal shown at the "a" and "b" were generated by wet-ozonization of P400.

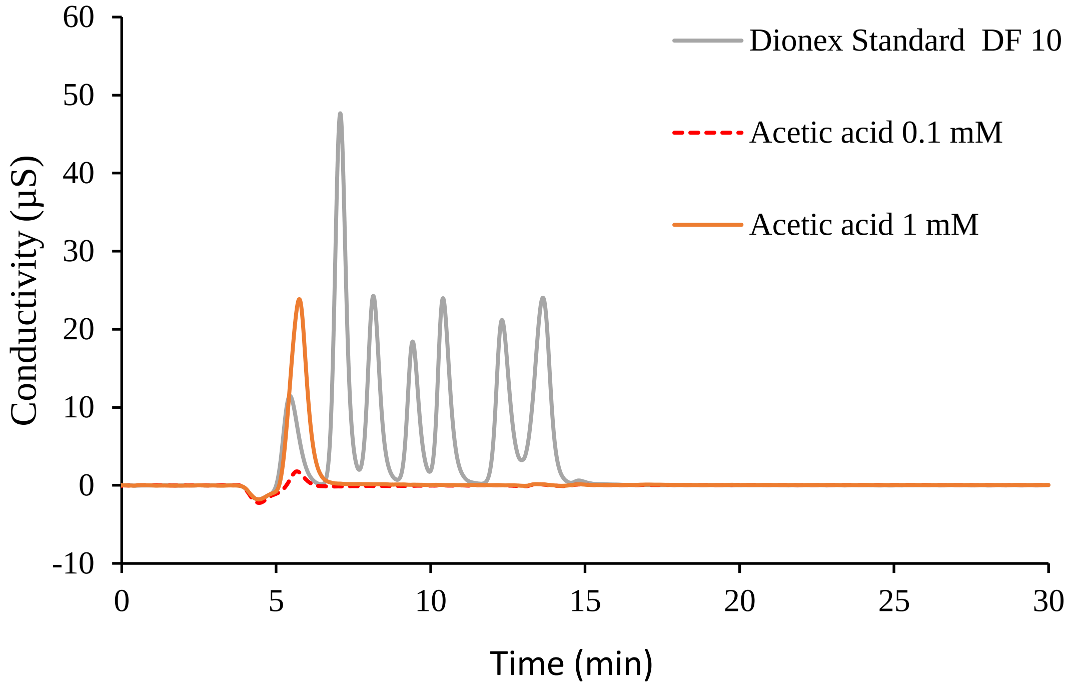

Figure S16: Conductivity signals of standard acetic acid. The standard Dionex seven (fluoride, chloride, nitrite, bromide, nitrate, phosphate, and sulfate) signals are also shown as a reference.

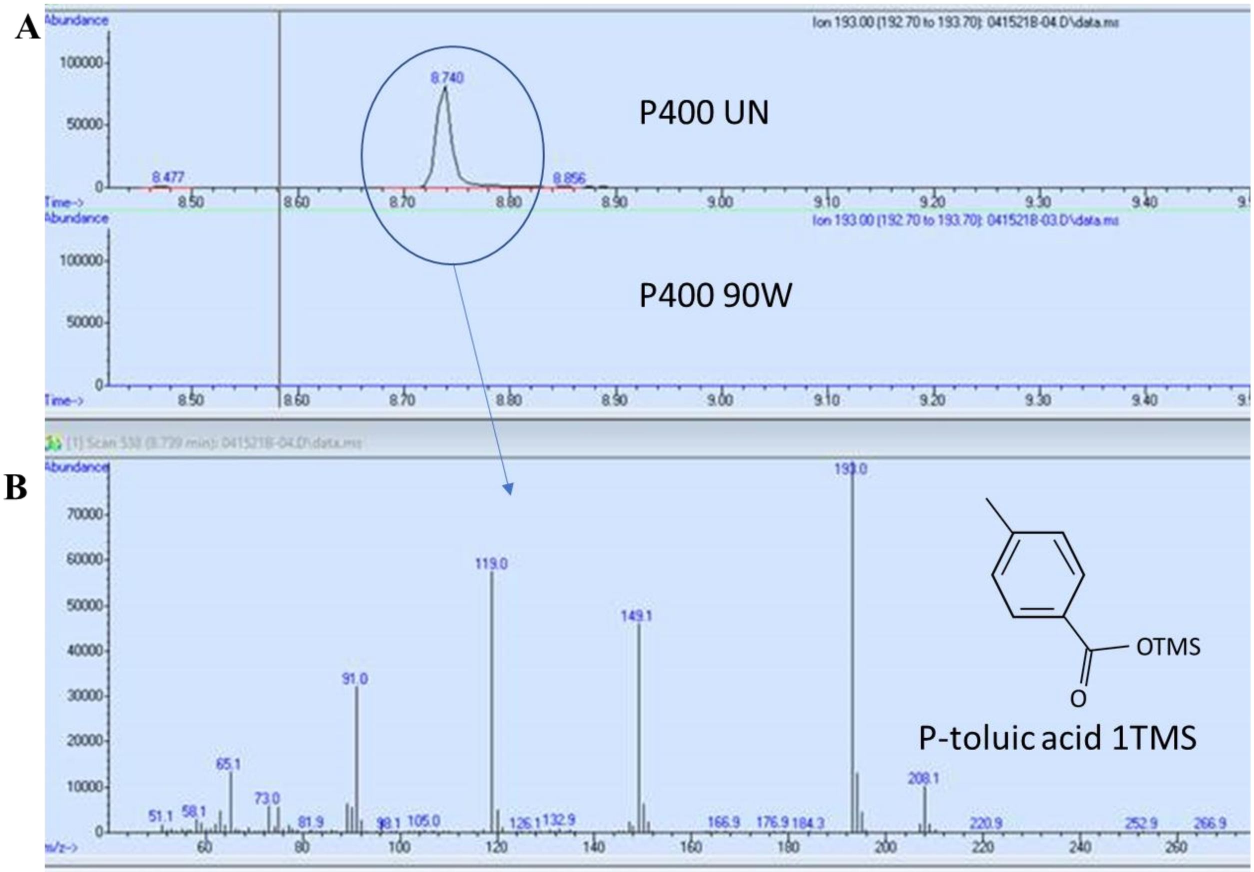

Figure S17: GC-MS spectra of the pine 400 biochar filtrate. In A; GC spectra of the filtrate from non-ozonized pine 400 (P400 UN) on the top graph and wet-ozonized pine 400 (P400 90W) on the bottom graph. The mass spectra of the signal detected in P400 UN (circled) is shown in B. In B; mass spectra of compound (p-toluic acid 1TMS or trimethylsilyl) detected in the GC spectra of P400 UN.

A

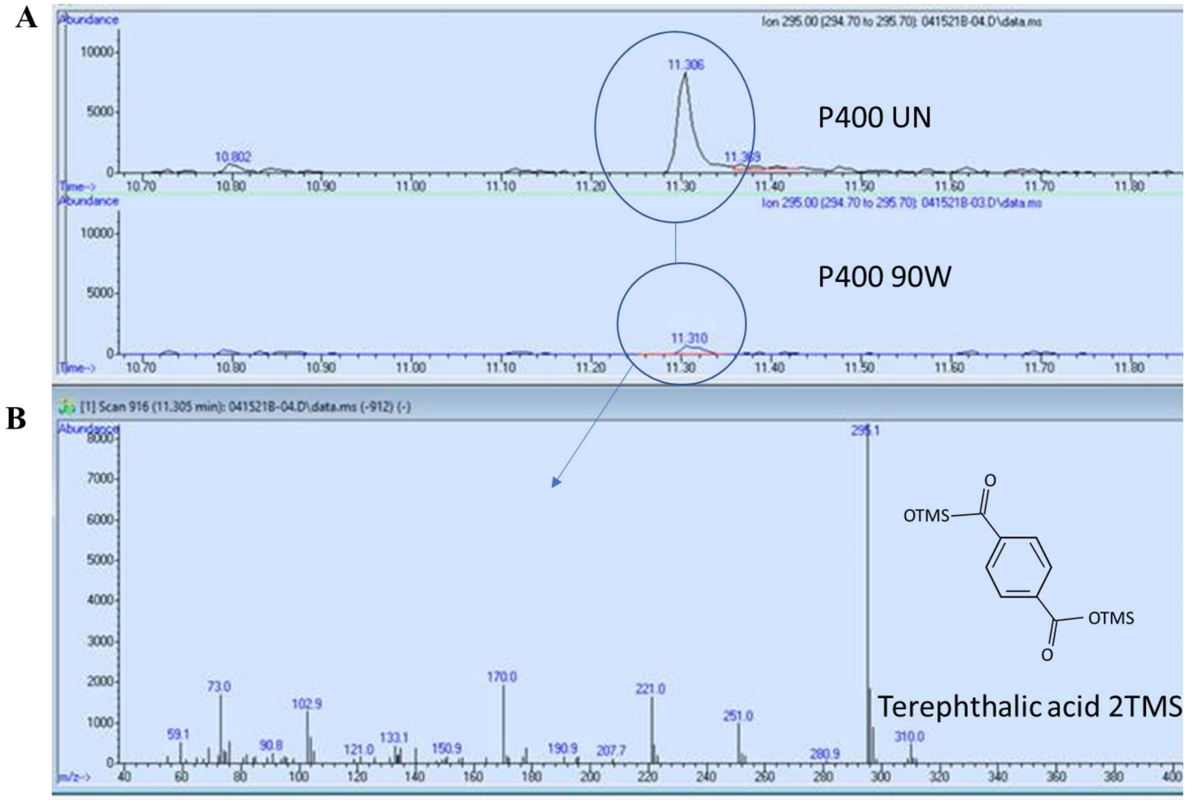

Figure S18: GC-MS spectra of the pine 400 biochar filtrate. In A; GC spectra of the filtrate from non-ozonized pine 400 (P400 UN) on the top graph and wet-ozonized pine 400 (P400 90W) on the bottom graph. The mass spectra of the signal detected in P400 UN and P400 90W (circled) is shown in B. In B; mass spectra of compound (terephthalic acid 2TMS or trimethylsilyl) detected in the GC spectra of P400 UN and P400 90W.
